# The Impact of COVID-19 on Adult Day Services' Closures and Programming

**DOI:** 10.1093/geroni/igab046.422

**Published:** 2021-12-17

**Authors:** Lauren Parker, Katherine Marx, Joseph Gaugler, Laura Gitlin

**Affiliations:** 1 Johns Hopkins Bloomberg School of Public Health, Baltimore, Maryland, United States; 2 Johns Hopkins School of Nursing, Baltimore, Maryland, United States; 3 University of Minnesota, Minneapolis, Minnesota, United States; 4 Drexel University, College of Nursing and Health Professions, Drexel University, Pennsylvania, United States

## Abstract

Nationally, adult day services (ADS) were forced to closed due to the COVID-19 pandemic. The forced closure of ADS programming consequentially impacted the services provided to clients. Many ADS continued to provide telephonic/remote services to clients, despite limited reimbursement from national and state sources for these services. Using data from ADS sites participating in the ADS-Plus Program (n= 22), this presentation examines the effects of COVID-19 on ADS closures and programming provided during the closure. About 86% (n=19) of the centers reported having to closed due to COVID-19. One-hundred percent of the sites reported offering telephone support to clients. Nearly 45% (n=10) of the centers reported not being reimbursed for this service. As ADS is a vital community-based resource for many families, it is important to demonstrate the crucial services provided by ADS to inform policymakers of the essentiality of day centers.

